# Hints and Improvements in the Practice of Medicine, Surgery, and Pharmacy

**Published:** 1800-01

**Authors:** 


### HINTS AND IMPROVEMENTS

### IN THE PRACTICE OF

### MEDICINE, SURGERY, AND PHARMACY.

---

### *On the Medicinal Effects of the Carbonat of Potafs, or Salt of Tartar, in Puerperal Fevers: by Citizens* ALLAN, DEYEUX, GILBERT, *et* LAFISSE.

[Extracted from a Memoir of Cit. GUINOT, inferted in the xxxvii Number of the Recueil Periodique de la Société de Medecine de Paris.]

CITIZEN GUINOT divides this important paper into two diftinct fections: the firft contains the theory on which the author has founded his practice; and the fecond confifts of eight obfervations on cafes felected from a number of patients whom he had under his care.

He obferves, that being little fatisfied with the principal works publifhed on puerperal fever, he has for a long time employed himfelf in making new inquiries into this fubject. The refult of feveral experiments has induced him to confider the real caufe of this dreadful difeafe to be *a predominance of acid.*

1. He took the urine of women attacked with puerperal fever, and diluted it with diftilled and filtrated water. It imparted to the folution of turnfol a deeper red than that produced by the fame dye, mixed with the urine of a healthy perfon, or of a woman recently delivered without accident. Thefe experiments being feveral times repeated, uniformly gave a fimilar refult.

2. He obferved, that in puerperal fever an ichor fometimes flows through the vagina, which has an acid fmell, fimilar to that of milk which is beginning to turn four.

3. The humour partly lactiform, and partly cafiform, found in the abdomen of women who have died of puerperal fever, has likewife an acid fmell; and, when diluted with diftilled and filtrated water, it gives a red colour to the folution of turnfol, which leaves no doubt of its acidity.

The abovementioned experiments induced the author to imagine that alkali would either neutralize the fuperabundant acid, or prevent its formation. Having learnt that LEVERET fuccefsfully fully ufed the carbonat of potafs, internally as well as externally, againft metaftafes of the milk formed under the fkin, he thought the fame means might be advantageoufly employed in fuch lacteous metaftafes as happen in the abdomen, or other parts, in puerperal fevers.

Wifhing to be more confirmed in his opinion, the author triturated the cafeous fubftance which commonly covers the exterior furface of the inteftines of women who die of the difeafe alluded to, with a given quantity of the carbonat of potafs, and obferved that fubftance adopt the appearance of a milky fluid. He alfo tried white cheefe, as well as moft other cheefes known at Paris; by adding water and the carbonat of potafs, they were completely diffolved. Having mixed feveral forts of milk with this alkaline fubftance, he boiled them and left them expofed to the heat of the fun, without being able to make them curdle; in which, however, he immediately fucceeded, when he repeated the experiment with milk, unmixed with the carbonat of potafs.

Being thus convinced that puerperal fever arofe from a fuperabundance of acid, and that the carbonat of potafs would be an excellent means of cure, Cit. Guinot, after feveral fuccefsful experiments, exhibited from ten to thirty-fix grains a day, not only in puerperal fever, but in all difeafes connected with the fecretion of milk in the female breaft.*

He alfo employed foaps and alkalis externally, without neglecting other remedies indicated by the circumftances and fymptoms of the cafe.

[ To be continued in our next Number. ]

---

* The effects of the falt of tartar in difeafes of child-bed women are well known, both in England and Germany. Induced by the example of feveral phyficians on the continent, Dr. W. has frequently prefcribed from 25 to 30 drops of *Ol. Tart. per deliquium*, to be taken two or three times a day, in a cup of ftrong mutton or veal broth, with fingular fuccefs. This remedy often afforded fpeedy relief from tingling pains in the breafts of young mothers who were nurfing their firft or fecond children, and had either mifmanaged, or neglected to fecure their breafts in cold weather. He has alfo recommended it in a late cafe of a woman who was troubled with callous and painful breafts from external injury, fo that fhe was apprehenfive of an incipient cancer. Here the exhibition of the abovementioned medicine afforded more effectual relief from pain than opiates, though it did not eventually remove the complaint.

### *On a new Species of Salt, and its Use in the Treatment of several Diseases. By Cit.* CHAUSSIER.

[Abftracted from the Recueil Periodique, No. xxxvii. p. 23 and feq.]

AFTER fome general reafons advanced by Cit. Chauffier, to prove that fulphur deferves much attention in the practice of medicine, not only becaufe that fubftance is found in the membranes and fluids of organized bodies, and is the caufe of feveral phenomena; but alfo, as it furnifhes feveral compofitions which are ufed with advantage both in the arts, and in the treatment of difeafes, he prefented to the Society a new fpecies of falt, which differs confiderably from the fulphats, fulphites, and fulphurs. The properties of this falt are very remarkable, from the ftate and nature of its component fubftances, as well as from the manner in which they are united.

This falt is a combination of hydrogenated fulphur with foda; in which the fulphur is found in confiderable proportion, and the hydrogen ferves as the medium of union, while it in fome degree imparts the property of an acid.

The new falt is formed fpontaneoufly in manufactories where, with a view to obtain foda for exportation, the fulphat of foda, or Glauber's falt, is decompofed according to the procefs fuggefted by Citizens MALHERBE, ATHENAS, and ALBAN. This procefs is adopted in the manufactory of Cit. PAYEN, and confifts in melting, in a reverberatory furnace, the fulphat of foda with a certain quantity of carbon and filings of iron. As we cannot at prefent give our readers the whole courfe of this procefs, we fhall content ourfelves with ftating, that the fulphurated hydro-fulphur of foda is found in the refiduum of the leys, when they no longer yield the carbonat of foda by ebullition. As thefe mother-leys ftill contain a certain quantity of cauftic foda, they are collected and preferved in the manufactory with the intention of extracting from them the remaining fulphat of foda. After ftanding for fome time, a cryftallization more or lefs abundant is formed in the midft of the alkaline refiduum of thefe leys; but the cryftals are not a carbonat of foda, as might at firft be fuppofed; nay, they are entirely different in their form as well as their compofition. Much care fhould be taken not to mix them with the foda already prepared; as they render it impure, give it a fulphureous fmell, and make it unfit to be ufed in feveral pharmaceutical and other preparations. In fhort, this new falt is the fulphurated hydro-fulphur of foda. As, in the firft cryftallization, the falt is yellow, and foiled, or tinctured by a blackiſh powder attached to its ſurface, it is neceſſary to diſſolve it in a certain quantity of water, and after it is filtered, and nearly diſſolved, it is put in a cool place to cryſtallize. Thus purified, this ſalt is diſtinct from all others in many of its properties, of which the following are the principal:

1. It forms large, brilliant, colourleſs, and tranſparent cryſtals in the form of a quadrangular priſm, terminated by an hexagonal pyramid.

2. It does not change the blue, violet, or yellow colours of teſt papers; hence it is a perfectly neutral ſalt.

3. It has a freſh taſte, which ſoon becomes ſlightly bitter, and leaves in the mouth a ſenſation like that imparted by hydrogenated ſulphur, which remains for ſome time.

4. When expoſed to the action of air, it preſerves its form and properties; it neither crumbles into powder nor deliqueſces: in ſhort, it does not change into a ſulphat, as is the caſe with ſalts formed by the combination of the ſulphureous acid with an alkaline baſis.

5. A cryſtal of this ſalt placed upon red hot coals quickly melts, bubbles, becomes dry, and at length burns with a blue flame, which emits a ſulphureous ſmell.

6. It is inſoluble in alcohol; a ſort of cryſtallization may be inſtantly obtained, by pouring alcohol on an aqueous ſolution of this ſalt.

7. Cold water diſſolves nearly thrice its weight of this ſalt: the ſolution is limpid and colourleſs.

8. If cryſtals of this ſalt be expoſed to the fire in a retort, they melt in temperature of 38° of the new *Thermomètre centigrade.*

9. If the cryſtals of this ſalt be diſtilled in an apparatus with mercury, the whole produce carefully collected, and the atmoſpheric air excluded, a certain quantity of water is firſt obtained; a portion of ſulphur is afterwards ſublimed, and a ſulphuret of ſoda remains in the retort, which, like other combinations of this kind, is ſoluble in water, changes when in contact with air, but during the whole operation no gas is diſcharged.

10. This ſalt decompoſes metallic ſolutions, and forms two new compounds. The ſolvent acid combines with the ſoda, and the hydrogenated ſulphur unites with the metallic ſubſtance.

11. All acids decompoſe this ſalt, by diſplacing its ſoda which forms an eſſential part; but ſome of them in their action preſent peculiar phænomena.

A. If ſulphuric acid be poured on the cryſtals of this ſalt, the mixture almoſt immediately boils; there is likewiſe heat and a diſcharge of ſulphurated hydrogen gas, and ſulphureous acid gas; there at length remains a ſulphat of ſoda, incruſtated with a yellow, flexible, and ſoft pellicle of ſulphur.

B. A diſcharge of hydrogen gas and ſulphureous acid likewiſe takes place, when the ſulphuric acic is poured on an aqueous ſolution of this ſalt; and the ſulphur alone is precipitated in a very fine powder.

C. Similar reſults are obtained, with little difference, by the nitric and muriatic acids.

D. The ſulphureous acid decompoſes this ſalt, and precipitates a ſulphur.

E The acetous, tartarous, boracic, and oxalic acids, arſenic, &c. alſo decompoſe this ſalt, and precipitate a ſulphur, but without cauſing any ſenſible diſcharge of gas. Some of theſe decompoſitions preſent phænomena, which Cit. Chauſſier propoſes to publiſh at a future period. We ſhall only remark, that according to him the gaſes obtained by the affuſion of acids, are not always the ſame, but are in proportion to the greater or leſs affinity which the acid bears to water, or to the facility with which it is decompoſed; and though by the affuſion of ſome acids a diſcharge of ſulphureous acid gas is obtained, and this ſalt is immediately formed, as has been already ſtated, by combining a ſulphite of ſoda with ſulphurated hydrogen, &c. yet, when the combination is made, no acids any longer exiſt, at leaſt they cannot be diſcovered by theſe proceſſes which appear the moſt certain. In ſhort, if a ſmall quantity of a ſolution of barytes in water is poured into an aqueous ſolution of this ſalt, no precipitation, or perceptible change takes place, except that in a few ſeconds the liquor grows yellowiſh, which indicates a new combination of ſulphur; the liquor becomes alkaline, and reddens paper prepared with curcuma. If a larger quantity of the water of barytes is poured on, in a few minutes there is a ſaline cryſtallization formed, which adheres to the ſides of the veſſel, and the liquor becomes more and more alkaline. At length the ſalt is entirely decompoſed; the barytes combines with the hydrogenated ſulphur, and the ſoda, pure and cauſtic, remains diſſolved in the water.

12. From theſe and analogous experiments it is aſcertained, that the component parts of this new ſalt are in the following proportion:

| | | |
|---|---|---|
| Hydrogenated ſulphur — — | 34 | 9 |
| Soda — — — — | 24 | 6 |
| Water — — — — | 40 | 5 |
| | 100 | |

Cit.

Cit. Chauſſier has employed this ſalt with advantage in the treatment of inveterate herpetic affections, which were not accompanied with fever, or inflammation; it may alſo be preſcribed in ſome diſeaſes of the viſcera, ariſing from metaſtaſes, or the repulſion of a pſoric and ſcorbutic virus.

It may be preſcribed for internal uſe, in doſes of from 30 to 40 decigrammes, either diſſolved in water, or made up in pills. As this ſalt contains ſulphurated hydrogen, it may be uſed with advantage as a ſubſtitute for the ſulphureous mineral waters, which are brought at a great expence from diſtant places, and conſequently loſe their efficacy. It may be employed in ſhower baths, lotions, or potions.

The internal uſe of this ſalt ſhould always be commenced with ſmall doſes, and gradually increaſed. Cit. Chauſſier preferred the uſe of it in pills of three decigrammes, or about ſix grains, made of the crumb of bread, and ordered ten or eleven to be taken every morning and evening. He alſo mentions that he cured, by this remedy, a venereal affection, attended with two exoſtoſes, flying pains, and a great number of ſpots and puſtules on the ſkin.

The efficacy of this remedy is promoted by ptisans, and other drinks proper for the patient. In ſhort, it is with this as with other medicines, which are only efficacious according to the method in which they are employed, the proper time of their application, and the aid which their operations receive from regimen.

This ſalt is ſold at the Manufactory of Cit. Payen, and at Cit. Pelletiers, Apothecary, in Jacob-ſtreet.

